# Split luciferase complementation assay to detect regulated protein-protein interactions in rice protoplasts in a large-scale format

**DOI:** 10.1186/s12284-014-0011-8

**Published:** 2014-06-28

**Authors:** Yukichi Fujikawa, Takahiro Nakanishi, Hiroko Kawakami, Kanako Yamasaki, Masa H Sato, Hiroyuki Tsuji, Makoto Matsuoka, Naohiro Kato

**Affiliations:** 1 Graduate School of Biosphere Science, Hiroshima University, 1-4-4 Kagamiyama, Higashi-Hiroshima 739-8528, Hiroshima, Japan; 2 Faculty of Human Environmental Sciences, Kyoto Prefectural University, Kyoto 606-8522, Japan; 3 Department of Plant Biology, Graduate School of Biological Sciences, Nara Institute of Science and Technology, 8916-5 Takayama, Ikoma 630-0192, Nara, Japan; 4 Bioscience and Biotechnology Center, Nagoya University, Nagoya Aichi 464-8601, Japan; 5 Department of Biological Sciences, Louisiana State University, 226 Life Sciences Building, Baton Rouge 70803, LA, USA

**Keywords:** Interactome, Split luciferase complementation, Regulated protein-protein interactions

## Abstract

**Background:**

The rice interactome, in which a network of protein-protein interactions has been elucidated in rice, is a useful resource to identify functional modules of rice signal transduction pathways. Protein-protein interactions occur in cells in two ways, constitutive and regulative. While a yeast-based high-throughput method has been widely used to identify the constitutive interactions, a method to detect the regulated interactions is rarely developed for a large-scale analysis.

**Results:**

A split luciferase complementation assay was applied to detect the regulated interactions in rice. A transformation method of rice protoplasts in a 96-well plate was first established for a large-scale analysis. In addition, an antibody that specifically recognizes a carboxyl-terminal fragment of *Renilla* luciferase was newly developed. A pair of antibodies that recognize amino- and carboxyl- terminal fragments of *Renilla* luciferase, respectively, was then used to monitor quality and quantity of interacting recombinant-proteins accumulated in the cells. For a proof-of-concept, the method was applied to detect the gibberellin-dependent interaction between GIBBERELLIN INSENSITIVE DWARF1 and SLENDER RICE 1.

**Conclusions:**

A method to detect regulated protein-protein interactions was developed towards establishment of the rice interactome.

## Background

Interactome analysis collects data of protein-protein interactions occurring in cells. A useful application of the analysis is to identify functional modules of signal transduction pathways in which a network of protein-protein interactions mediate the signals (Barabasi et al. [2011]; Pawson and Nash [2000]). Protein-protein interactions occur in cells in two ways, constitutive and regulative. Constitutive protein interactions occur all the time in cells. In many cases, even when interaction is examined *in vitro*, a constitutive interaction is detectable. On the other hand, regulated protein interactions occur when cells are exposed to selected environments such as stress and environmental cue. When cells are exposed to selected environments, some of proteins change their subcellular localization to interact with other proteins. Alternatively, cells may produce small compound (i.e., hormone) that mediates a protein-protein interaction (Ritter and Hall [2009]), or a protein in the cells be modified (i.e., phosphorylation) to attract other proteins (Pawson [2004]). Importantly, regulated protein-protein interactions are often at pivotal positions within the interaction network in a signal transduction pathway (Wehr et al. [2008]).

Because plants highly adaptive to different environments, signal transduction pathways in plants are thought to be complicated (Vanstraelen and Benkova [2012]; Santner and Estelle [2009]). Genomes of *Oryza* genus have been modified to adapt diverse environments and increase the value as a crop (Vaughan et al. [2003]). Hence, genetic analysis of *Oryza sativa*, rice, is recently spurred considerable interest to understand the genomic evolution. However, rice interactome analysis, which would reveal the molecular mechanism of the rice adaptation, has been limited to constitutive protein-protein interactions that are determined by the yeast two-hybrid assay (Gu et al. [2011]; Seo et al. [2011]). Although the assay would detect protein-protein interactions in absent or present of certain regulators, it is difficult to detect regulated protein-protein interactions that may occur in cellular conditions specific to rice.

The split luciferase complementation (SLC) assay is one of methods that detect regulated protein-protein interactions *in situ* (Ozawa et al. [2001]; Kaihara et al. [2003]; Luker et al. [2004]). In the assay, N- and C-terminal fragments of luciferase are genetically fused to a protein pair of interest. The luciferase activity, which emits light through oxidation of a substrate, is complemented when the protein pair interacts with each other, but ceases when the protein pair does not interact. Hence, one can determine a protein interaction by a flash of light. Because the assay is capable of identifying not only interaction but also dissociation of protein pairs, it is suitable for analyzing kinetics of protein interactions *in situ* (Luker et al. [2004]). Moreover, the result of the SLC assay is reproducible with high signal-to-background ratios, which allows for conducting interactome analysis, at least, in animal cells (Cassonnet et al. [2011]).

In plants, the assay was first applied to detect a histone-histone interaction in protoplasts of Arabidopsis leaves (Fujikawa and Kato [2007]). Since then, the assay has been applied to detect interactions between membrane proteins (Kato et al. [2009]), bacterial effector proteins and their protein targets (Chen et al. [2008]), auxin response factors (Li et al. [2011]), 14-3-3 regulator proteins (Gehl et al. [2011]), coiled-coil–nucleotide-binding site–leucine-rich repeat (CC-NB-LRR) protein (Inoue et al. [2013]), and kinase (Schmidt et al. [2013]). We previously showed with the SLC assay that protein-protein interactions in Arabidopsis protoplasts are sensitive to environmental conditions (Kato and Bai [2010]), indicating that the SLC assay would be well-suited for analyzing regulated protein-protein interactions in plant cells.

Currently, however, the use of the SLC in plants is limited to detecting constitutive protein-protein interactions and only in dicot model plants such as Arabdidopsis and tobacco, but not in monocot plants such as rice. Comparative transcriptional analyses revealed that rice and Arabidopsis differently transduce a stress signal (Yazaki et al. [2004]; Narsai and Whelan [2013]). Moreover, gene annotation and transcriptome analyses revealed that rice and Arabidopsis differently regulate the flow of potassium in the plasma membrane as a result of stress and environmental cue (Very et al. [2014]; Ma et al. [2012]). Potassium is the major inorganic cation in the cytoplasm, and known to regulate the activities of many enzymes including ones involved in signal transductions in plant cells (Wang and Wu [2013]). These analyses support the idea that rice may possess a unique network in regulated interactions, which could not be identified when a protein-interaction assay is conducted in different organisms (i.e. Arabidopsis and yeast).

Here we present a *Renilla* luciferase-based technology breakthrough that allows the identification of regulated protein-protein interactions in rice in near-real time, which can also be expanded to a large-scale analysis.

## Results and discussion

### A transformation method for rice protoplasts in a 96-well plate was established

**Figure 1:**
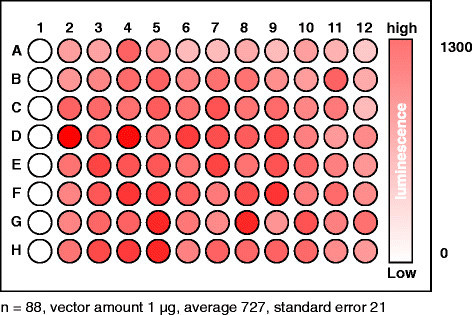
**A method to transform rice protoplasts in a 96-well plate.***Renilla* luciferase activities in rice protoplasts transformed with pYFP-RLuc, the vector expressing full-length *Renilla* luciferase (Fujikawa and Kato [2007]) were measured in a 96-well plate. Protoplasts (5,000 cells) isolated from rice leaves were placed in each well, except in wells of column 1 that served as sample-free controls. The protoplasts were then transformed with the vector. The luminescence in each well was measured by a microplate reader 16 h after the transformation. The relative luminescence units (RLUs) in each well are expressed as a white-to-red heatmap. The calibration bar is shown on the right. Notice transformation bias is not observed in the plate. n: sample number.

Simultaneously transforming an organism of interest with a large number of independent vectors is one of the key technologies for an interactome assay. Although methods to transform rice protoplasts in individual micro-centrifuge tubes have been published elsewhere (Bart et al. [2006]; Zhang et al. [2011]; Datta and Datta [1999]), a method simultaneously to transform rice cells in a 96 well-plate hat allows transforming the cells with a large number of independent vectors has not been established for rice. To this end, we established a method in which rice protoplasts are transformed with polyethylene glycol (PEG) in a 96-well plate, based on the method previously developed for Arabidopsis leaf protoplasts (Kato and Jones [2010]). We used a vector expressing full-length *Renilla* luciferase as a transgene so that the transformation method was most optimized for the assay. We found that 0.5 – 1.0 g of 1 – 2 week old rice shoots that are vertically cut into 2 mm wide strips toward the leaf sheath produced enough high quality protoplasts to conduct 96 independent transformations in the plate. We also found that transforming the rice protoplasts in a 96-well plate did not generate a bias toward column and row in the plate with respect to transformation efficiency (Figure 1). In other words, transformation efficiency is slightly deviated in each well but not by columns and rows.

### An antibody that recognizes the C- terminal fragment of *Renilla* luciferase was generated

**Figure 2:**
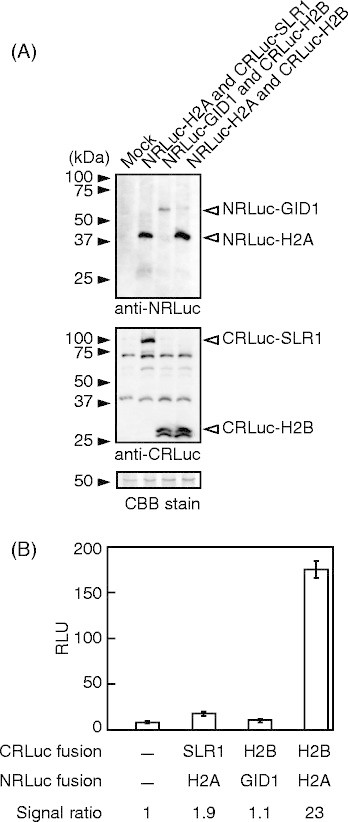
**The interaction of histone-2A and histone-2B was detected by complemented luciferase activity in rice protoplasts. (A)** Accumulations of split luciferase fused to histone 2A (NRLuc-H2A), GID1 (NRLuc-GID1), histone 2B (CRLuc-H2B), or SLR1 (CRLuc-SLR1) in rice protoplasts. The cell extracts were analyzed by immunoblot assays using anti-NRLuc or anti-CRLuc antibodies. Equal protein loading was confirmed by coomassie brilliant blue (CBB) staining. **(B)** Luminescence signals in rice protoplasts expressing combinations of NRLuc-H2A, NRLuc-GID1, CRLuc-H2B or CRLuc-SLR1 in a 96-well plate. The graph shows the mean RLU ± S.E. (n = 3). The accumulated proteins and signal ratio are described at the bottom of the graph. Notice only protoplasts expressing NRLuc-H2A and CRLuc-H2B show RLU that is significantly higher than that of the non-transformed protoplasts.

It is important to understand quality and quantity of recombinant proteins accumulated in transformed protoplasts. This ensures interaction kinetics is correctly interpreted from luminescence emitted from the protoplasts. We previously found that a commercially available anti-*Reniila* luciferase antibody recognizes the N-terminal fragment (NRLuc) but not the C-terminal fragment (CRLuc) of *Renilla* luciferase (Fujikawa and Kato [2007]). Thus, a polyclonal antibody that recognized CRLuc was generated. The antibody was directed against GST-fused CRLuc. To test whether the antibody recognizes recombinant proteins properly, rice protoplasts were transformed with vectors expressing recombinant proteins (Figure 2). The recombinant proteins expressed were *Arabidopsis* HISTONE 2A (H2A, AT4G27230), *Arabidopsis* HISTONE 2B (H2B, AT5G22880), rice GIBBERELLIN INSENSITIVE DWARF1 (GID1, Os05g0407500), or rice SLENDER RICE 1 (SLR1, Os03g0707600). The cDNAs encoding these proteins were inserted in pDuEx vectors (Fujikawa and Kato [2007]). These vectors contain NRLuc or CRLuc, and express the recombined gene from the cauliflower mosaic virus 35S promoter (Fujikawa and Kato [2007]; Kato and Jones [2010]). Recombinant proteins were expressed in rice protoplasts in various combinations (Figure 2). To conduct immunoblot assays for evaluating the *Renilla* luciferase antibodies, proteins in the transformed rice protoplasts were extracted by lysing the cells 16 h after the transformation. The proteins were then electrophoresed and transferred to a membrane in order to blot with the newly generated anti-CRLuc antibody, or the commercially available antibody that recognizes NRLuc. The results revealed that both antibodies detect recombinant proteins accumulated in rice protoplasts (Figure 2A). Also, the recombinant proteins did not degrade much because signal bands in the immunoblot were not smeared. The anti-CRLuc antibody detected multiple proteins whose sizes are between 40 and 75 kDa, which do not correspond to the CRLuc-fused proteins. This indicated that careful analysis would be required when recombinant proteins whose sizes are similar to those unknown proteins are examined. It also suggested that levels of protein accumulations would depend on a protein expressed. For Instance, the accumulation level of the NRLuc-GID1 protein is lower than that of the NRLuc-H2A protein (Figure 2A).

Luminescence emitted from protoplasts depends on not only the affinity (dissociation constant) of a protein pair that is fused to NRLuc and CRLuc, respectively, but also the amounts the recombinant proteins accumulated in the protoplasts (Kato et al. [2009]). Because luminescence units determined in the assay are relative but not absolute, it remains a challenge to deduce an absolute value of the dissociation constant. However, the anti-CRLuc antibody developed in this study will allows addressing the question whether low luminescence in the assay is caused by a low affinity of the protein pair or low levels of the protein accumulation in one or both of the protein pair as discussed in the following sections.

### Histone2A-histone2B interaction was detected with a high signal-to-background ratio

To examine whether a protein interaction was detected by bioluminescence in rice protoplasts, relative luminescence units (RLU) were measured soon after adding the luciferase substrate to the culture solution in each well of the 96-well plate but before lysing the protoplasts for the immunoblot assay. Because H2A interacts with H2B (Fujikawa and Kato [2007]), we expected rice protoplasts expressing NRLuc-H2A and CRLuc-H2B to emit luminescence light at high levels upon addition of viviRen®, the substrate for *Renilla* luciferase. On the other hand, we expected rice protoplasts expressing NRLuc-H2A and CRLuc-SLR1 or NRLuc-GID1 and CRLuc-H2B to emit luminescence light at very low levels, similar to mock-transformed protoplasts, because these protein pairs would not interact with each other. The rice protoplasts expressing NRLuc-H2A and CRLuc-H2B showed 21-fold higher RLU, compared to the mock transformed protoplasts (Figure 2B). On the other hand, the protoplasts expressing NRLuc-H2A and CRLuc-SLR1 or those expressing NRLuc-GID1 and CRLuc-SRL1 showed RLU at low levels relative to the mock transformed protoplasts. This suggested that rice protoplasts are suitable to determine protein-protein interactions by the SLC assay in a large-scale format as we previously found in *Arabidopsis* protoplasts (Fujikawa and Kato [2007]).

### Gibberellin-dependent interaction of GID1 and SLR1 was detected in near-real time

Adaptation of plant growth and development to the environment is largely achieved through hormone production and distribution in the plant (Vanstraelen and Benkova [2012]; Santner and Estelle [2009]). Hence, signal transduction pathways and a protein interaction network in plant cells are expected to be modulated by hormones. To test whether our system could detect hormone-dependent protein interactions, we took advantage of the previously characterized interaction between two components of gibberellin signaling, GID1 and SLR1 (Ueguchi-Tanaka et al. [2005]).

GAs are a large family of plant hormones that widely play roles in plant development, such as seed germination, stem elongation, leaf expansion, flowering, and pollen maturation (Thomas and Sun [2004]). A breakthrough in the GA signaling study is the discovery of a soluble GA receptor, GID1 (Ueguchi-Tanaka et al. [2005]). The *gid1* mutant rice shows dwarf and insensitive to the exposure of exogenous GA. Structural analyses of GID1 proteins of rice and Arabidopsis revealed that an N-terminal portion of the protein functions as a lid to bind and cover GA (Murase et al. [2008]; Shimada et al. [2008]). The N-terminal lid is also found to involve in the GA-dependent interaction with SLR1, the repressor in GA signaling (Murase et al. [2008]; Shimada et al. [2008]). Interestingly for us, while the rice (*Oryza sativa*) genome encodes one GID1 protein OsGID1, the Arabidopsis (*Arabidopsis thaliana*) genome encodes the OsGID1 orthologous protein AtGID1a and the paralogous proteins AtGID1b (Nakajima et al. [2006]). AtGID1b constitutively interacts with SLR1 without GA (Yamamoto et al. [2010]). This supports the idea that rice and Arabidopsis cells may have different networks in the GA-regulated protein-interactions.

**Figure 3:**
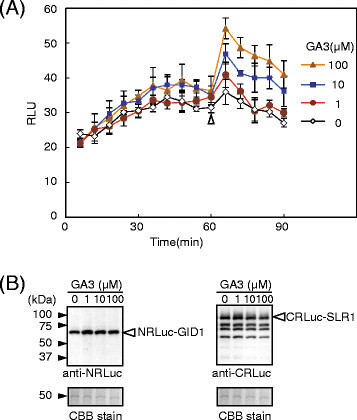
**GA3-concentration dependent GID1-SLR1 interaction was detected in rice protoplasts. (A)** Rice protoplasts were transformed with a pair of vectors expressing NRLuc-GID1 and CRLuc-SLR1 in a 96-well plate. Luminescence signals were measured about every 6 min for 90 min before and after adding gibberellin (GA3, 1, 10, 100 μM) or W5 buffer (as a negative control) in rice protoplasts. GA3 and W5 buffer were added at 60 min after adding luciferase substrate (indicated with an open triangle). The graph shows mean RLU ± S.E. (n = 6). **(B)** Proteins were extracted from the transformed protoplasts. The proteins were analyzed by immunoblot assays using the anti-NRLuc antibody (left panel) or anti-CRLuc antibody (right panel). Equal protein loading was confirmed by CBB staining. Notice the amounts of recombinant proteins do not change.

Because the interaction of GID1 and SLR1 occurs in the nucleus in a gibberellin-dependent manner in rice (Ueguchi-Tanaka et al. [2005]; Ueguchi-Tanaka et al. [2007b]), we tracked changes of RLU in rice protoplasts transformed with vectors expressing NRLuc-GID1 and CRLuc-SLR1 (Figure 3). In the experiment, different concentrations of gibberellin 3 (GA3), an active form of gibberellin, were added to the culture solution 60 min after adding the luciferase substrate. The protoplasts showed low RLU (about 20 RLU) soon after adding the substrate, suggesting interaction did not occur at this point. Although the RLU gradually increased with a function of time, most likely due to passive transport of the substrate from the culture solution into the cells, the RLU remained at low levels for 90 min without GA3. On the other hand, the RLU increased soon after adding GA3, and the RLU remained at high levels even 30 min after adding GA3 (90 min after adding the substrate). Fold changes of the RLU after adding GA3 (up to 1.5-fold, compared to the mock treated protoplasts) was not as high as the H2A-H2B interaction that also occurs in the nucleus (10-fold, compared to a non-interaction protein pair) (Figure 2). This suggested that the dissociation constant of GID1-SLR1 might be lower than that of H2A-H2B. An additional or alternative possibility is that topologies of NRLuc-GID1 and CRLuc-SLR1 do not allow for complementation of the luciferase activity to the extent of that with NRLuc-H2A and CRLuc-H2B. In other words, three-dimensional positions of NRLuc and CRLuc may not be optimized to reconstitute the luciferase activity when NRLuc-GID1 and CRLuc-SLR1 interact. Nevertheless, the increased RLU was statistically significant (P < 0.01) and depended on the concentration of GA3. Moreover, GA3 did not increase RLU in the protoplasts that expressed NRLuc-H2A and CRLuc-H2B (Additional file 1: Figure S1). We also confirmed by immunoblot assay that the changes of the RLU were not due to changes of the amounts of the recombinant proteins accumulated in the transformed protoplasts (Figure 3B). Together the results indicated that the increased RLU in the protoplasts expressing NRLuc-GID1 and CRLuc-SLR1 was due to increased association of the recombinant proteins by GA3.

### *Interaction of GID1 and SLR1 was not affected by other plant hormones in rice protoplasts*

**Figure 4:**
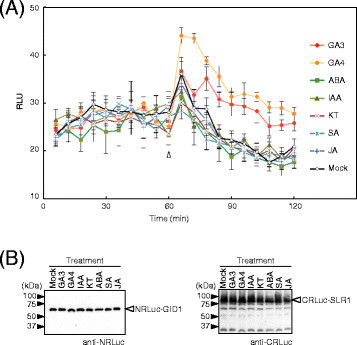
**The GID1-SLR1 interaction is specific for gibberellin in rice protoplasts. (A)** Rice protoplasts were transformed with a pair of vectors expressing NRLuc-GID1 or CRLuc-SLR1 in a 96-well plate. Luminescence signals were measured about every 6 min for 120 min before and after adding gibberellin 3 (GA3), gibberellin 4 (GA4), abscisic acid (ABA), indoleacetic acid (IAA), kinetin (KT), salicylic acid (SA), and jasmonic acid (JA) at 10 μM and W5 buffer (as a negative control) in different wells. The plant hormones and W5 buffer were added at 60 min after adding the luciferase substrate (indicated with an open triangle). The graph shows the mean RLU ± S.E. (n = 6). **(B)** Proteins were extracted from the transformed protoplasts. The proteins were analyzed by immunoblot assays using the anti-NRLuc antibody (left panel) or anti-CRLuc antibody (right panel). Equal protein loading was confirmed by CBB staining. Notice the amounts of recombinant proteins do not change by the plant hormones in this condition.

Gibberellin signaling pathways exhibit crosstalk with pathways that are regulated by other plant hormones such as abscisic acid and auxin (Weiss and Ori [2007]). To investigate whether other plant hormones besides gibberellin trigger the GID1-SLR1 interaction in rice cells, we tracked changes of RLU in the rice protoplasts transformed with vectors expressing NRLuc-GID1 and CRLuc-SLR1 (Figure 4). In the experiment, GA3 and GA4, another active form of gibberellin, as well as other plant hormones, including abscisic acid (ABA), indoleacetic acid (IAA), kinetin (KT), salicylic acid (SA), and jasmonic acid (JA) added to the culture solution 60 min after addition of the luciferase substrate. RLU increased soon after adding GA3 and GA4, and the RLU remained at high levels even 30 min after adding GA3 and GA4 (90 min after adding the substrate). Fold changes of the RLU, compared to the mock transformed protoplasts, after adding GA4 was significantly (P < 0.01) higher than that of GA3. This suggested that GID1 and SLR1 might interact more firmly with GA4 than GA3. This agrees with a previous report that GA4 gives higher promoter activity in a GID1-SLR1 yeast two-hybrid assay compared to GA3 (Ueguchi-Tanaka et al. [2007a]).

Although other hormones increased RLU, the increases were not significant (P > 0.10) compared to the mock transformed protoplasts. We also confirmed by immunoblot assays that amounts of the recombinant proteins accumulated in the transformed protoplasts were not affected by different plant hormones (Figure 4B). This suggested that the GID1-SLR1 interaction was mediated by gibberellin specifically in the rice cells. This also showed that the SLC assay is capable of detecting a protein-protein interaction that is regulated by a hormone.

## Conclusions

We previously discussed about advantage and disadvantage of SLC by comparing the results of SLC in Arabidopsis protoplasts with that of other interaction assays such as FRET (Fluorescence resonance energy transfer) and Co-IP (Co-immunoprecipitaiton) assay (Fujikawa and Kato [2007]; Kato et al. [2009]). We, furthermore, used the SLC assay to detect a light-dependent interaction in Arabidopsis protoplasts (Kato et al. [2009]). Here we showed that the SLC is capable of detecting a protein-protein interaction that is regulated by a hormone in rice protoplasts in near-real time. Because pDuEx vectors used in this SLC assay contain the Gateway® recombinant sites (Fujikawa and Kato [2007]), one can relatively easily construct a large number of expression vectors. Moreover, four different types of pDuEx vectors that allow fusing NRLuc or CRLuc to N- or C-terminal ends of a target protein, were constructed (GenBank: EF565883, EF56588, GU370778 and GU370779). All together, these provide a suite of vectors for a large-scale SLC assay in rice protoplasts. The suite would aid in revealing regulated protein-protein interactions in rice and other monocot crop cells in a large-scale format.

## Methods

### Plasmid constructs

The coding sequences in the cDNAs including a stop codon (GID1; GIBBERELLIN INSENSITIVE DWARF1, Genbank Acc. No. AB211399, SLR1; SLENDER RICE1 Genbank Acc. No. AB262980) were amplified by PCR using pGBKT7-GID1 and pGADT7-SLR1 (Ueguchi-Tanaka et al. [2005]) as the templates, respectively. The products of PCR were then cloned into a pDONR/Zeo vector (Invitrogen, CA). The resultant plasmids were subsequently used to clone into pDuExAn6 or D7 expression vectors (Fujikawa and Kato [2007]) with the Gateway® cloning system (Invitrogen, CA).

### Rice cultivation and treatments

Mature seeds of rice (*Oryza sativa* L. Japonica) were soaked in water for 3 days at 28°C in the dark after being sterilized with 50% (v/v) bleach for 10 min. The seeds were then placed on soil in a pot (8 cm wide by 7.5 cm high) with Kimura B solution [365 μM (NH_4_)_2_SO_4_, 547 μM MgSO_4_, 183 μM KNO_3_, 365 μM Ca(NO_3_)_2_, 182 μM KH_2_PO_4_, 19 μM Fe-EDTA, 48.7 μM H_3_BO_3_, 9 μM MnSO_4_, 0.3 μM CuSO_4_, 0.7 μM ZnSO_4_, 0.099 μM Na_2_MoO_4_] containing 1 nM uniconazole. The seedlings were incubated at 28°C in 16 h of light (light intensity; 100 μmol m^−2^ sec^−1^) and 8 h of dark for 1 to 2 weeks.

### Protoplast preparation and transformation

Rice shoots (0.5 - 1.0 g, 1–2 week old) were cut into 2 mm-wide sections toward the leaf sheath by using a surgical knife (No.21 FETHER, Osaka, Japan). Protoplasts were isolated by enzymatic hydrolysis using cellularase RS (Yakult pharmaceutical industry. Tokyo, Japan) and macerozyme R10 (Yakult pharmaceutical industry). The pieces of the rice shoots were first incubated with the enzyme solution (4% cellulase RS and 2% macerozyme R10, 0.6 M mannitol, 20 mM MES pH 5.7, 20 mM KCl, and 10 mM CaCl_2_) for 4 h on a rotary platform shaker at 35 rpm/min after vacuuming the samples for 30 min. The samples were then filtered through a 40 μm nylon mesh to remove clumps. The isolated protoplasts were washed two times with 10 ml of W5 buffer (0.125 M CaCl_2_, 5 mM KCl, and 2 mM MES pH 5.7) by centrifugation at 200 × g for 3 min. The protoplasts were further suspended in 10 ml of W5 buffer and placed at 4°C for 30 min. The protoplasts were collected by centrifugation at 200 × g for 3 min, and re-suspended in MMg solution (0.4 M mannitol, 15 mM MgCl_2_, and 4 mM MES pH 5.7) so that the concentration of the protoplasts in the solution would be 4 × 10^5^ cells/ml. Protoplast transformation was conducted using a 96-well plate (U-bottom plate; U96 MicroWell Plates, Nalge Nunc International, NY) based on the method previously published (Kato and Jones [2010]). Briefly, the expression vectors dissolved in 10 μl of distilled water were used to transform 1.6 × 10^4^ protoplasts in 40 μl of the MMg buffer. After adding 60 μl of polyethylene glycol solution containing 40% (w/v) PEG4000 (Sigma-Fluka, MO), 0.2 M mannitol and 0.1 M CaCl_2_ to the wells, the plate was vortexed with a digital vortex mixer (GENIE2, Scientific industries) at 900 rpm for 15 sec. The transformed protoplasts in each were then washed with 200 μl of W5 buffer. To replace the solution in each well, the plate was centrifuged at 200× g for 3 min at room temperature and 200 μl of the supernatant was removed from each well. The remaining protoplasts were washed four-times in 200 μl of W5 buffer. The transformed protoplasts (1.6 × 10^4^ cells) were suspended in 100 μl of W5 buffer. Finally, the plate was shaken for 5 sec and incubated in the dark at 28°C overnight.

### Measuring luciferase luminescence

Luminescence was measured by a microplate luminometer (ARVOx4 2030 Multilabel Reader, Perkin Elmer, MA) immediately after adding 10 μl of 0.12 mM ViviRen® (Promega) in each well where the transformed protoplasts were incubated in the 100 μl solution. The luminescence signals in each well were integrated for 0.5 sec in one measurement every 1.5 min. When hormones were added, luminescence signals in a 96-well plate were measured about every 6 min for 90 min or 120 min before and after adding plant hormones. GA3 and GA4 were purchased from Sigma-Fluka. Abscisic acid (ABA) and jasmonic acid (JA) were from Wako Pure Chemical (Osaka, Japan). Indoleacetic acid (IAA), kinetin (KT) and salicylic acid (SA) were from Nakarai tesque (Kyoto Japan).

### Generation of antibody

cDNA encoding the C-terminal fragment of *Renilla* luciferase was amplified from pDUExD7 (Fujikawa and Kato [2007]) by PCR using CRLuc-F (5’-CCGGAATTCAAGCCCGACGTCCAGATT-3’) and CRLuc-R (5’-CCGCTCGAGCTGCTCGTTCTTCAGCACGCG-3’) primers. The resulted PCR product was subcloned into pGEX5X-1 (GE Healthcare, WI). The resultant plasmid was introduced into BL21, and the GST-fused CRLuc protein fragment was induced by adding 0.1 mM IPTG for 12 hr at 30°C. The fusion protein was purified by glutathione Sepharose 4B according to the manufacture’s instruction (GE Helthcare). The purified protein was used to obtain a polyclonal antibody using rabbit.

### Immunoblot analysis

Rice protoplasts (3.2 × 10^4^ cells) suspended in 10 μl of W5 buffer were homogenized with 10 μl of 2x SDS-PAGE sample buffer containing 0.2 M Tris–HCl (pH 6.8), 20% (v/v) glycerol, 4% (w/v) SDS, 0.15 M sodium chloride, 2% (v/v) Triton X-100, 0.005% (w/v) brome phenol blue, 4% (v/v) 2-mercaptoethanol, and 6 M urea. The samples were boiled for 5 min and loaded on a 5-20% gradient polyacrylamide gel (E-R520L, Atto Corp., Tokyo, Japan). Proteins in the gel were then transferred to a polyvinylidene difluoride membrane (Immobilon-P transfer membrane, Millipore Corp., MA) using a semidry electrotransfer (15 V constant). The membrane was incubated for 90 min in a blocking solution [4% (w/v) nonfat dry milk and 0.1% (v/v) Triton X-100 in 10 mM Tris–HCl, pH 7.4, 150 mM sodium chloride), and further incubated overnight with mouse monoclonal anti NRLuc (1:20,000, MAB4400, Millipore Corp) or anti-CRLuc rabbit serum (1:10,000) in the blocking solution. The binding of these antibodies was detected with an ECL Western blotting system (Amersham Bioscience/GE Healthcare, WI).

## Competing interests

The authors declare that they have no competing interests.

## Authors' contributions

FY, NT and KH carried out the experiments. FY also drafted the manuscript. MHS and KY participated generated an antibody. HT and MM participated in constructing expression vectors for rice genes. NK conceived of the study, and participated in its design and coordination, and drafted the manuscript. All authors read and approved the final manuscript.

## Authors' information

FY was a postdoctoral associate in LSU at the early stage of the study.

## Additional file

## Supplementary Material

Additional file 1: figure S1. Gibberellin does not affect the H2A-H2B interaction. Click here for file
