# Supplementary material for: Split luciferase complementation assay to detect regulated protein-protein interactions in rice protoplasts in a large-scale format
Source: Rice (N Y). 2014 Jun 28;7(1):11. doi: 10.1186/s12284-014-0011-8 (PMC4077619; doi:10.1186/s12284-014-0011-8)
Supplement: Additional file 1: figure S1. — Gibberellin does not affect the H2A-H2B interaction. [file s12284-014-0011-8-S1.docx]

**Supplemental Figure S1. Gibberellin does not affect the H2A-H2B interaction.**

Rice protoplasts were transformed with a couple of the vectors expressing NRLuc-H2A and CRLuc-H2B, respectively, in a 96-well plate. Luminescence signals in the plate were then measured about every 6 min for 120 min before and after adding GA3 (10 µM) in the protoplast suspensions. GA3 was added at 60 min after starting measuring (indicated with an open triangle). The figure shows the mean RLU ± S.E. (n=6).
